# Characterization of Unpublished Manuscripts by Applicants to an Orthopedic Hand Surgery Fellowship

**DOI:** 10.1016/j.jhsg.2023.08.010

**Published:** 2023-09-29

**Authors:** Jennafir Ernst, Mark E. Baratz, John R. Fowler

**Affiliations:** ∗ West Virginia University, Morgantown, WV; † University of Pittsburgh Medical Center, Pittsburgh, PA

**Keywords:** Fellowship, Hand Fellowship, Hand surgery, Orthopedic hand

## Abstract

**Purpose:**

Obtaining a hand surgery fellowship is becoming increasingly competitive, and research is an important factor when assessing applications. Given the competitive nature of the fellowship application process, applicants may feel the need to bolster their application by misrepresenting their research experience. One form of misrepresentation rarely discussed in prior studies is the listing of submitted works under a “Publications” heading in curricula vitae. This study examines the prevalence of misclassification of manuscripts by applicants to a hand surgery fellowship and identifies factors that might be associated with incorrect classification.

**Methods:**

A retrospective review of 122 applicants to the 2020–2021 cycle for hand surgery fellowship was performed. Names and identifiable information were redacted prior to review. Demographic data collected included sex, United States Medical Licensing Examination Step 1 score, medical school rank, residency specialty, total publications, presence of submitted manuscripts in the “Publications” section, total number of submitted manuscripts, and total published abstracts and poster presentations.

**Results:**

A total of 1,098 listed publications across the 122 applicants were reviewed with a median of five publications per applicant. Submitted manuscripts were listed as publications by 33 applicants (27%). No observable differences by age, United States Medical Licensing Examination Step 1 score, or total number of publications were seen. Misclassification rates were not associated with publication totals.

**Conclusions:**

More than one-quarter of applicants incorrectly listed submitted or unaccepted manuscripts as publications. It is our hope that making fellowship applicants aware of this issue will decrease the rates of misrepresentation in future application cycles.

**Clinical relevance:**

The competition for hand surgery fellowships has become more intense, and this may explain our finding that 27% of applicants misrepresent the status of research on hand surgery fellowship applications.

Misrepresentation of research in applications for medical residency and fellowship programs is a relatively common occurrence, and there is some evidence that the frequency may be increasing.^1^^,^^2^ Changes to the Electronic Residency Application Service (ERAS) in 2014, specifically the inclusion of the PubMed Identifier might account for much lower rates of misrepresentation in recent years.^3^ Research publications are one of several criteria used to evaluate orthopedic residency applications and was ranked as the fourth most important factor in a survey of orthopedic fellowship program directors.^4^^,^^5^ The importance of research publications in the evaluation of applicants to hand fellowship programs may result in either purposeful or inadvertent inflation of research activities.

Rates of research misrepresentation vary widely by publication year, study design, and definition of misrepresentation.^1^^,^^6^, ^7^, ^8^, ^9^, ^10^ A meta-analysis suggested misrepresentation occurs in 4.9% of applications for both residency and fellowship based on the strictest definition. This remains heavily dependent on how researchers define misrepresentation. Wiggins^6^ noted in a meta-analysis that many studies did not consider self-promotion in authorship or listing a more prestigious journal as publisher to be misrepresentation. However, there was nearly universal agreement in prior studies that ghost publications or non-authorship is a misrepresentation of work. While nonexistent articles and elevation of authorship are clearly unprofessional and unethical, prior studies have included other minor misrepresentations to include incorrect page numbers, titles, or misspellings.^11^

One form of misrepresentation rarely discussed in prior studies is the listing of submitted works under a “Publications” heading in a curriculum vitae. Publication rates for submitted manuscripts on residency applications range from 43.7% to 67.5%, and those that are published are frequently in lower impact journals than initially presented at the time of application submission as noted by Freshman et al.^12^^,^^13^ The goal of this study was to review and present the frequency of hand fellowship applicants listing submitted manuscripts under a “Publications” heading and to identify any applicant factors that might be associated with incorrect classification when applying to a hand surgery fellowship.

## Materials and Methods

### Data collection

A retrospective review of 122 applications to a single hand surgery fellowship program from the 2020–2021 application cycle was completed. Data extracted from each application included United States Medical Licensing Examination (USMLE) Step 1 score, applicant sex, attendance at a Top 20 US News & World Report Medical School, residency specialty, curriculum vitae sections, total publications, presence of submitted manuscripts in the “Publications” section, total number of submitted manuscripts, and total published abstracts and poster presentations. Total publications included accepted or in-press listings. For the purpose of this study, these were not verified as having been published due to the recency of submission and relatively high publication rates.^14^

### Statistical analysis

All statistical analyses were performed using SPSS 20. Descriptive statistics were used for demographical data. The Shapiro–Wilk test was used to evaluate the normality of data distribution. Comparison of group membership (ie, those who listed submitted publications as publications and those who did not) was conducted using Mann-Whitney U test and Pearson’s chi-square test. A binary logistic regression was completed to examine all variables (eg, age, sex, Top 20 US News Rankings medical school, Step 1 score, and total publications) to group membership. Statistical significance was defined as *P* <.05.

## Results

### Applicant demographics

There were a total of 122 applicants to our hand surgery fellowship for the 2020–2021 cycle. Demographics for these applicants are summarized in Table 1. The mean applicant age was 31.5±2.5 years with a mean USMLE Step 1 score of 246±13. Sixty-six percent of applicants were male. Ten applicants (8.2%) were from a Top 20 medical school. Applicant residency specialty was predominately orthopedic surgery (83%) followed by plastic surgery (12%) and general surgery (5%).

**Table 1:** Demographic, USMLE Step 1 Score, and Publication Data by Group

	Correctly Classified (n = 89)	Misclassification (n = 33)	*P Value*
Age	31.5 ± 2.6	31.6 ± 2.2	.39
Sex (% male)	57/89 (64%)	23/33 (69.7%)	.56
USMLE step 1 score	247 ± 13.8	244 ± 12.6	.42
Top 20 school	8/89 (9%)	2/33 (6.1%)	.73
Total publications	10.25 ± 20.7	5.64 ± 5.7	.30
Published abstracts and presentations	5.7 ± 7.5	6.8 ± 6.2	
Total publication≤1	14/89 (15.7%)	10/33 (30.3%)	.08

USMILE, United States Medical Licensing Examination.

### Publication characteristics

There were a total of 1,098 listed publications across the 122 applicants with a mean of 9±18. A Shapiro–Wilk test showed publications were not normally distributed (*P* <.001), and nonparametric tests were used to compare groups (Table 1). Median number of publications per applicant was five publications. Ten percent of applicants had no publications. Curricula vitae analysis revealed a large majority of applicants (n = 95; 77.9%) listed a stand-alone section of “Peer-Reviewed Publications,” “Publications,” or “Published Manuscripts.” Remaining applicants listed a combined research section (eg, “Publications, Abstracts, and Presentations”). Submitted manuscripts were listed as publications by 33 applicants (27%). The number of submitted manuscripts listed as publications ranged from one to nine per applicant, with approximately half (n = 17) listing one or two. There were no observable differences by age (*P* =.39), Step 1 score (*P* =.42), or total number of publications (*P* =.30). Although the mean publication total for the correct classification group (10.3) was twice that of the misclassification group (5.6), this was the result of two outliers with over 100 publications, and median scores for each group were equivalent (5.0 for each group). Furthermore, applicants who listed a submitted manuscript as a publication were not less likely to have zero publications (*P* =.64) or fewer than ten publications (*P* =.16) than their colleagues.

## Discussion

In a sample of applicants for a hand surgery fellowship, over three-quarters correctly differentiated published manuscripts from unpublished submitted works on their curricula vitae while approximately one-quarter listed submitted or not yet accepted manuscripts as publications*.* It is our hope that making fellowship applicants aware of this issue will decrease the rates of misrepresentation in future application cycles. The most likely explanation is that applicants are attempting to bolster or improve the perception of their research section during application. Although one might expect this to occur more frequently with applicants who have low publication totals, the results of this study did not support that hypothesis. Likewise, a prior review of neurosurgery residency applicants found applicants with more publications were more likely to commit misrepresentations on application to residency.^2^ Another possible explanation is that applicants were unaware of how to properly list ongoing research projects. Indeed, prior studies have found medical students have limited knowledge of subjects such as authorship etiquette.^15^ Since nearly all of these applicants previously completed a residency application that provided a structured breakdown of research activities as well as the same rate of misrepresentation occurring on high-publication curricula vitae*,* this explanation seems less likely.

There is limited agreement on what constitutes misrepresentation but often includes ghost publications, incorrectly listing self as first author, and listing a more prestigious journal than published, among others.^6^ As the ERAS application system has evolved, other types of misrepresentation have emerged, such as listing non-peer–reviewed articles or online only articles under the peer-reviewed section of the application.^2^ These prior studies have noted the inherent dishonesty in elevating the status of a publication to a level of scrutiny it did not receive. Given that the 2014 changes to the ERAS may have contributed to the decreased misrepresentation rate on residency applications, incorporating a similar standardized classification system for fellowship could reduce the rates seen among these applications as well. We suggest that future studies include the listing of unpublished, unaccepted submitted manuscripts under a “Publications” heading on a curriculum vitae to be a form of misrepresentation. There may also be benefit to a multisite examination of acceptance rates of applicants based on research representation; however, that is beyond the scope of this work.

There are several limitations to this study. First, it is a single year sample, and the degree to which this represents an outlier year has not been established. Second, this is a retrospective review, and there are inherent limitations. Although this is a relatively large group to study, a subsequent multiyear review with more applicants included may help elucidate subtle group differences or other factors associated with these misrepresentations not seen in this analysis.

To date, there have been a number of studies that have examined many types of misrepresentation from fabricated publications to typos and incorrectly listed journals. One such study conducted by Freshman et al^13^, while similar in content, examined medical students applying to orthopedic surgery residency programs and observed the likelihood of eventual publication of submitted works. The current study similarly looked at submitted/published research but utilized a different level of training (medical residents applying for fellowship). The purpose of this study was also different, specifically examining how applicants identified their work by placing submitted works under “Publications” to demonstrate research productivity when the submitted work had not yet been published. This current study builds on these prior studies by examining how these submitted works are characterized by applicants and differences in applicant characteristics at the fellowship level.

The purpose of this study is to examine the rates at which hand surgery fellowship applicants incorrectly classify submitted manuscripts on their curricula vitae*.* We believe this represents another kind of misrepresentation of research and is a form of minor academic dishonesty, whether intentional or not. Standardizing the fellowship application research section could reduce the rate of this and other forms of misrepresentation. Although no consensus appears to exist, the authors would suggest that the most appropriate place for unpublished submitted works would be under a heading separate from Publications, such as “Submitted Works” or “Current Projects.”
